# Polyisocyanide Hydrogels as a Tunable Platform for Mammary Gland Organoid Formation

**DOI:** 10.1002/advs.202001797

**Published:** 2020-07-26

**Authors:** Ying Zhang, Chunling Tang, Paul N. Span, Alan E. Rowan, Tilly W. Aalders, Jack A. Schalken, Gosse J. Adema, Paul H. J. Kouwer, Mirjam M. P. Zegers, Marleen Ansems

**Affiliations:** ^1^ Institute for Molecules and Materials Radboud University Heyendaalseweg 135 Nijmegen AJ 6525 The Netherlands; ^2^ Radiotherapy & OncoImmunology Laboratory Radboud University Medical Center Geert Grooteplein 32 Nijmegen GA 6525 The Netherlands; ^3^ Australian Institute for Bioengineering and Nanotechnology (AIBN) The University of Queensland Brisbane QLD 4072 Australia; ^4^ Experimental Urology Radboud University Medical Center Geert Grooteplein 32 Nijmegen GA 6525 The Netherlands; ^5^ Department of Cell Biology Radboud Institute for Molecular Sciences Radboud University Medical Center Geert Grooteplein 28 Nijmegen GA 6525 The Netherlands

**Keywords:** mammary glands, organoids, polyisocyanides, synthetic hydrogels, synthetic matrices

## Abstract

In the last decade, organoid technology has developed as a primary research tool in basic biological and clinical research. The reliance on poorly defined animal‐derived extracellular matrix, however, severely limits its application in regenerative and translational medicine. Here, a well‐defined, synthetic biomimetic matrix based on polyisocyanide (PIC) hydrogels that support efficient and reproducible formation of mammary gland organoids (MGOs) in vitro is presented. Only decorated with the adhesive peptide RGD for cell binding, PIC hydrogels allow MGO formation from mammary fragments or from purified single mammary epithelial cells. The cystic organoids maintain their capacity to branch for over two months, which is a fundamental and complex feature during mammary gland development. It is found that small variations in the 3D matrix give rise to large changes in the MGO: the ratio of the main cell types in the MGO is controlled by the cell–gel interactions via the cell binding peptide density, whereas gel stiffness controls colony formation efficiency, which is indicative of the progenitor density. Simple hydrogel modifications will allow for future introduction and customization of new biophysical and biochemical parameters, making the PIC platform an ideal matrix for in depth studies into organ development and for application in disease models.

## Graphical abstract

A fully synthetic and highly tailorable matrix material is presented that supports the formation of organoids. With minimal adhesive peptide functionalization, this polyisocyanide hydrogel allows single cells to develop into mammary gland organoids. As tuning the hydrogel properties controls organoid composition, future customization will set the stage for improved control of organoid production and in‐depth studies into organ development.

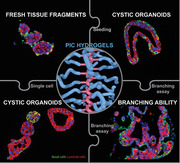

## 1 Introduction

The female mammary gland consists of a highly elaborate, tree‐like network of branched ducts and lobular alveolar structures, which are composed of an inner layer of luminal cells that surrounds a central lumen and of an outer layer of basal cells residing on the basement membrane. Over time, the mammary gland undergoes dramatic changes in structure and function during puberty, pregnancy, lactation and involution.^[^ ^1^, ^2^, ^3^ ^]^ The proliferative and differentiation potential retained by the adult mammary epithelium makes the mammary gland a unique model for biologists to study organ development^[^ ^4^, ^5^, ^6^, ^7^ ^]^ and disease generation.^[^ ^8^, ^9^, ^10^ ^]^ However, due to limitations in starting material, experimental accessibility and prolonged times during which development occurs, the mammary gland remains challenging to study in vivo.

The advent of stem cell‐derived 3D culture systems fills the gap between the complex organ in vivo and easily accessible but over simplified cell culture systems in vitro. They provide the scientific community with in vitro models that more closely represent in vivo physiology.^[^ ^11^, ^12^, ^13^ ^]^ The 3D structures derived from such stem cell‐derived 3D culture systems have been defined as organoids. During organoid formation, cells organize into structures that resemble the function and architecture of the corresponding native organ.^[^ ^14^ ^]^ As such, organoid technology promises exciting new insights into the fields of basic biology,^[^ ^15^, ^16^ ^]^ disease modeling,^[^ ^17^, ^18^, ^19^ ^]^ and regenerative medicine.^[^ ^20^, ^21^, ^22^ ^]^

For mammary gland studies, robust culture conditions that use matrices based on basement membrane extracts like Matrigel, supplemented with growth factors have been established to generate mammary gland organoids (MGOs) from mammary gland fragments or from single sorted mammary epithelial cells (MECs).^[^ ^23^, ^24^ ^]^ Studies on MGOs have offered unique insights into the mechanisms regulating cell fate,^[^ ^24^ ^]^ branching morphogenesis^[^ ^25^, ^26^ ^]^ and the critical role of the extracellular matrix (ECM) in mammary gland development.^[^ ^27^, ^28^ ^]^ Although animal‐derived matrices, such as Matrigel, support MGO formation and branching morphogenesis, their poorly defined composition and lot‐to‐lot variation hampers systematic investigation of the large role of ECM and biomechanics in organoid development. Moreover, the murine tumor‐derived matrices are incompatible with clinical transplantation. Only recently, the first synthetic extracellular matrices (ECMs), based on enzymatically crosslinked PEG hydrogels equipped with essential biological cues have been reported to replicate the natural ECM.^[^ ^29^, ^30^ ^]^ Such well‐defined ECMs constitute a powerful platform to identify biochemical and biophysical effects on stem cell‐derived organoid formation, branching morphogenesis and proliferation.^[^ ^31^, ^32^ ^]^

Here, we introduce a simple, yet highly versatile 3D culture system for murine MGOs based on synthetic hydrogels of oligo(ethylene glycol)‐grafted polyisocyanides (PICs).^[^ ^33^ ^]^ PIC hydrogels are a relatively new class of synthetic hydrogels that closely mimic the fibrous and porous architecture and mechanical properties of structural ECM proteins, such as collagen and fibrin. Gelation is thermally induced (heating a polymer solution beyond the gelation temperature *T*_gel_ ≈ 18 °C), very fast (within seconds) and fully reversible, which facilitates cell and organoid extraction.^[^ ^33^ ^]^ The hydrogels are physically crosslinked but stable over the course of cell culture experiments (weeks). Typically, the hydrogels that are formed at low polymer concentrations (0.1–1 wt%) are soft (shear modulus *G*′ = 0.1–4 kPa), which overlaps with the stiffness of mammary adipose tissue (NB: For Matrigel, *G*′ = 0.05 kPa). As a fully synthetic material, one can readily manipulate the network structure and mechanical properties by changing the polymer concentration, polymer molecular weight and external factors.^[^ ^33^, ^34^, ^35^, ^36^ ^]^ Recent work showed that PIC hydrogels are biocompatible^[^ ^37^, ^38^, ^39^ ^]^ and show no signs of toxicity during in vivo experiments.^[^ ^40^ ^]^ In these applications, PIC polymers are frequently functionalized with the well‐known cell adhesion peptide Gly‐Arg‐Gly‐Asp‐Ser (abbreviated to RGD), a motif that is found in many ECM proteins, including collagen, fibronectin, tenascin, and vitronectin.^[^ ^41^, ^42^ ^]^

In this manuscript, we demonstrate that PIC hydrogels only functionalized with the cell‐adhesive RGD peptide can support the generation of cystic MGOs from dissected mouse mammary gland fragments or from purified mouse mammary epithelial cells in vitro. The MGOs can be maintained for prolonged periods while retaining their capacity to branch. Moreover, we show that by independently tuning biomechanical and biochemical parameters (bulk stiffness and the peptide density), we are able to affect mammary gland progenitor enrichment and the cellular composition of the MGOs, respectively.

## 2 Results

### 2.1 Materials

**Figure 1:**
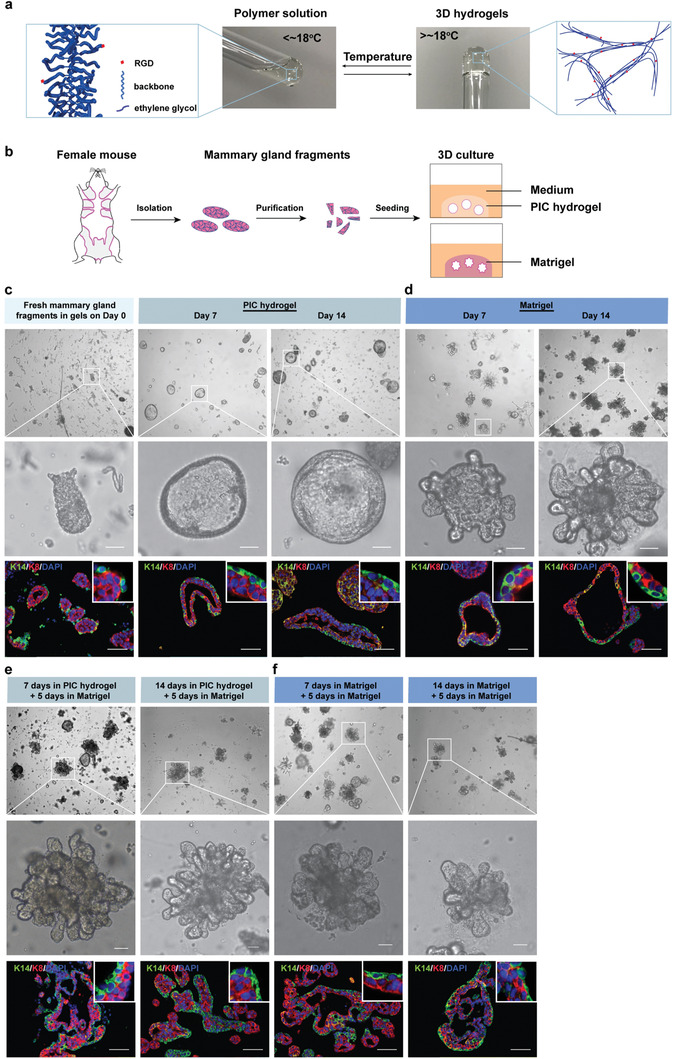
PIC hydrogels for mammary gland organoid formation. a) Aqueous PIC polymer solutions reversibly gel into a fibrous network by heating beyond ≈18 °C. b) Schematic description of the isolation and 3D culture of mammary gland fragments in PIC hydrogels or Matrigel. c,d) Representative bright‐field and immunofluorescence microscopy images of mammary gland fragments cultured in PIC hydrogels (0.2 wt%, 63 × 10^−6^ m RGD, *G*′ = 0.32 kPa, panel (c) or Matrigel (d) over time. Scale bars: 50 µm. e,f) Representative bright‐field and immunofluorescence microscopy images of PIC hydrogels (e) or Matrigel (f)‐derived MGOs after reseeding in Matrigel. Staining: luminal marker: rabbit anti‐cytokeratin 8 (K8, red); basal marker: mouse anti‐cytokeratin 14 (K14, green); and DAPI for nuclei (blue). Scale bars: 50 µm.

To prepare our matrix, we started from PICs functionalized with azide (N_3_) groups (**Figure** **1**a and Figure S1, Supporting Information),^[^ ^33^ ^]^ which can be reacted with acetylene‐equipped cell‐binding peptides (RGD) through the highly efficient strain‐promoted azide‐alkyne cycloaddition reaction.^[^ ^37^, ^38^, ^40^ ^]^ The matrix properties are tuned by independently modifying the polymer concentration, molecular weight and the RGD density; details are given in the Experimental Section. We first assessed the capacity of PIC hydrogels to support MGO formation in hydrogels from polymers of *M*_v_ = 502 kg mol^−1^ (3K) at a polymer concentration of 0.2 wt% (or 2 mg mL^−1^) and with an RGD peptide concentration of 63 × 10^−6^ m. Rheology experiments in HBSS give a gelation temperature *T*_gel_ = 18 °C and a storage modulus *G*′ = 0.3 kPa at 37 °C, corresponding to a Young's modulus *E′* ≈ 1 kPa, which is in line with mammary adipose tissue. Note that Matrigel is much softer than PIC hydrogels and mammary tissue: *G*′ ≈ 0.05 kPa. In a typical cell culture experiment, isolated cells or tissue fragments were mixed with cold PIC solutions (or Matrigel), pipetted in 24‐well plates and placed in an incubator at 37 °C to allow gel formation (0.5–1 h) and culture medium was placed on top (Figure 1b).

### 2.2 PIC hydrogels Support Organoid Formation from Mammary Gland Fragments

First, we assessed MGO formation in 3D PIC hydrogels from mammary gland fragments. To this end, freshly isolated murine mammary glands were minced into small fragments and seeded in PIC hydrogels or in Matrigel as a control (Figure 1b). To ensure homogeneous distribution in the gel matrix, the fragments were dispersed in a cold PIC solution (2 mg mL^−1^), warmed to 37 °C to induce gelation and overlaid with medium. Over seven days in culture, the mammary gland fragments in the PIC hydrogels changed shape, underwent self‐organization and expansion, and formed into bilayered structures with a central lumen which we will define here as cystic MGOs (Figure 1c). The fragments in the Matrigel control developed into bi‐ or multilayered branching structures with lumens, which we define here as branching MGOs (Figure 1d). Viability in both the PIC hydrogels and in Matrigel is high (>98%, Figure S2, Supporting Information).

The epithelial cell types present in the MGOs were characterized through immunostaining of the luminal epithelial cell marker keratin 8 (K8) and the basal/myoepithelial cell marker keratin 14 (K14).^[^ ^6^ ^]^ Co‐staining of K8 and K14 after seven days in culture revealed that MGOs generated in PIC hydrogels and in Matrigel both displayed the correct polarization with a K14‐positive basal outer cell layer and a K8‐positive luminal inner layer (Figure 1c,d). Previous work showed that the polarized organization of luminal epithelial cells around a central lumen critically depends on the interaction of its *β*1‐integrins with a basement membrane secreted by basal myoepithelial cells in vivo^[^ ^43^ ^]^ or provided by Matrigel in vitro.^[^ ^44^ ^]^ The positioning of luminal and basal cells around a central lumen of the cystic MGOs in the PIC‐RGD hydrogels strongly indicates that this gel promotes a proper assembly of a native basement membrane by basal/myoepithelial cells.

The cystic MGOs derived from PIC hydrogels did not form branches as observed in Matrigel. To assess whether the cystic MGOs retained branching capacity, they were harvested from the PIC solution (simply by cooling to 10 °C and washing) and then re‐embedded in Matrigel. After another five days in culture, the cystic MGOs showed extensive branching, similar as to MGOs solely cultured in Matrigel (Figure 1e,f). Immunofluorescence staining showed that these branching MGOs also consisted of an outer K14‐positive basal cell layer and an inner K8‐positive luminal cell layer. Moreover, both cystic MGOs generated in PIC hydrogels and branching MGOs derived from Matrigel could be maintained for at least 14 days without obvious deformation or loss in their branching capacity.

To demonstrate the versatility of this platform, prostate epithelial cells were seeded in PIC hydrogels and we observed that the PIC gel similarly supports the formation of cystic prostate organoids that are composed of luminal and basal cells that closely resemble the structures observed in Matrigel (Figure S3, Supporting Information).

The (mechanical) properties of PIC hydrogel are readily tuned by varying the polymer concentration and molecular weight;^[^ ^34^ ^]^ the latter has the advantage that this approach is easily synthetically accessible. We studied the effects of both parameters by culturing mammary gland fragments in different conditions and found MGO formation in all hydrogels (Figure S4, Supporting Information). Visual inspection suggests little differences between cultures of different molecular weight. hydrogels prepared at polymer concentrations *c* = 0.1% proofed too soft: part of the organoids sank to the bottom of the well and experience different culture conditions (Figure S5, Supporting Information). Note that the cells remaining in suspension have the same morphology as those seeded in higher PIC concentrations. Based on these results, we focused our study on PIC hydrogels with concentrations of 2–8 mg mL^−1^ (0.8 wt% approaches the solubility limit of PIC polymers) and with 0–250 × 10^−6^ m RGD.

### 2.3 PIC Hydrogels as a Matrix for Long‐Term Organoid Cultures

**Figure 2:**
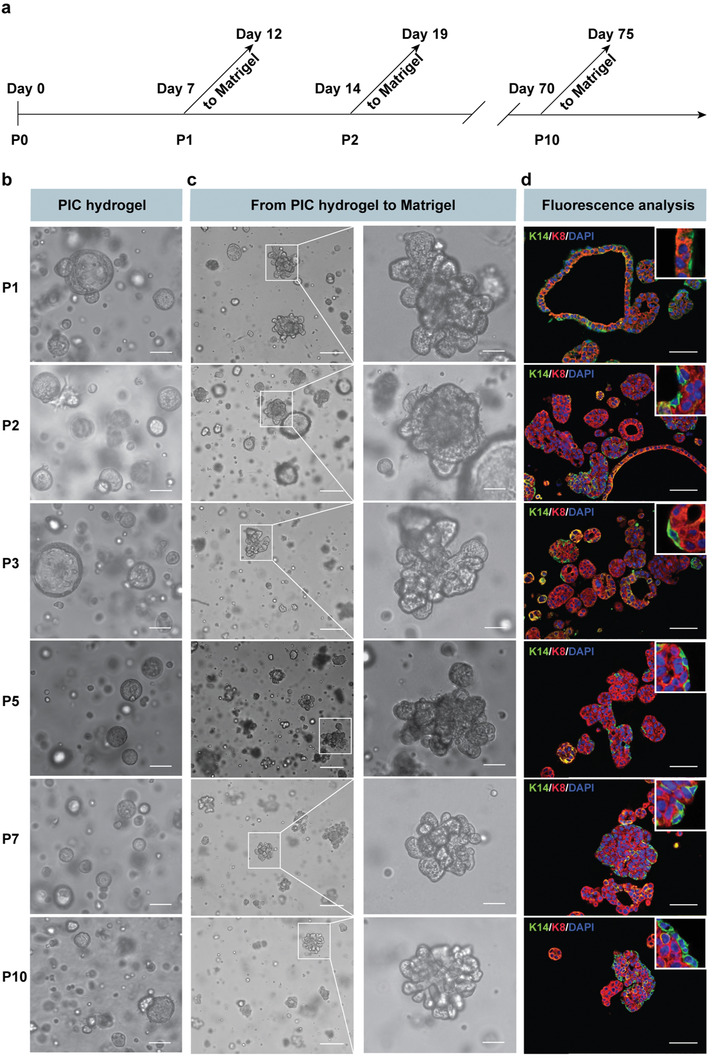
PIC hydrogels support MGOs long‐term maintenance. a) Schematic description of MGOs long‐term culture in PIC hydrogels (0.2 wt%, 63 × 10^−6^ m RGD, *G*′ = 0.32 kPa): after freshly isolated mammary gland fragments were cultured in PIC hydrogels for seven days, cystic MGOs were isolated from gels. A portion of MGOs was reseeded in Matrigel to study branching ability; another portion was enzymatically dissociated and reseeded in PIC hydrogels for long‐term passaging. Repeated passaging was carried out every week. b) Representative bright‐field images of MGOs generated in PIC hydrogels for ten passages. Scale bars: 50 µm. c,d) Representative bright‐field (c) and immunofluorescence (d) images of PIC hydrogels‐derived MGOs, reseeded in Matrigel. Scale bars: left panel 200 µm; right panels 50 µm. Staining: luminal marker: rabbit anti‐cytokeratin 8 (K8, red); basal marker: mouse anti‐cytokeratin 14 (K14, green); and DAPI for nuclei (blue).

Next, we assessed the potential of PIC hydrogels for long‐term MGO maintenance with retention of branching capacity (**Figure** **2**a). Again, freshly isolated mammary gland fragments were embedded in PIC hydrogels at the same conditions (2 mg mL^−1^ with 63 × 10^−6^ m RGD). After seven days, the hydrogels were cooled below the gelation temperature to harvest and isolate the MGOs, which were then enzymatically dissociated into single cells and/or very small cell clusters. Repeated passaging in PIC^[^ ^45^ ^]^ consistently yielded cystic MGOs (Figure 2b). When these cystic MGOs were re‐embedded in Matrigel, many branched extensively, but not all (Figure 2c). We observe that the cystic MGOs display K8‐positive cells only, while branching MGOs contain both K14‐positive basal and K8‐positive luminal cells (Figure 2d), which is a result of the dissociation into single cells as will be discussed below. We summarize that PIC hydrogels are well suited for long‐term cell cultures, where MGOs are formed consistently from single epithelial cells and where the organoids maintain their branching capacity.

### 2.4 MGO Formation from Purified Basal and Luminal Epithelial Cells

**Figure 3:**
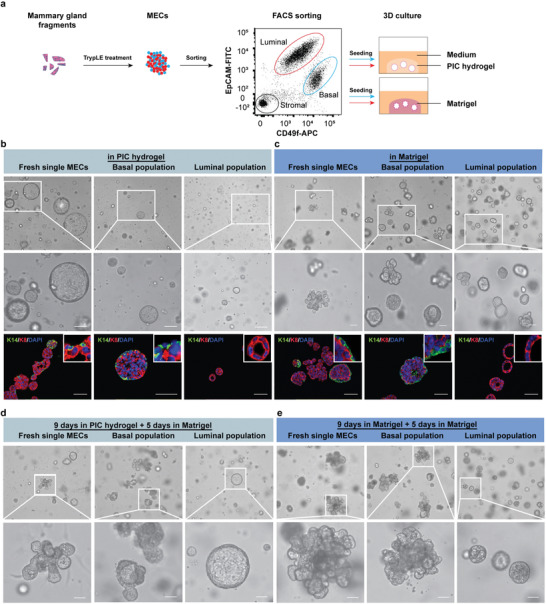
MGO formation in PIC hydrogels (0.2 wt%, 63 × 10^−6^ m RGD, *G*′ = 0.32 kPa) from mammary epithelial cells (MECs) and purified basal and luminal cells. a) Schematic description: fragments were digested to single MECs, FACS sorted into basal and luminal epithelial cells and then seeded in PIC hydrogels or Matrigel. b,c) Representative bright‐field and immunofluorescence images of MGOs generated from unsorted MECs (left), purified basal cells (middle) and luminal cells (right) in PIC hydrogels (b) or Matrigel (c) on day seven. Scale bars: 50 µm. d,e) Representative bright‐field images of MGOs generated from unsorted MECs (left), purified basal cells (middle) and luminal cells (right) in PIC hydrogels (d) or Matrigel (e) after reseeding in Matrigel. Staining: basal marker K14 (green), luminal marker K8 (red), and DAPI for nuclei (blue). Scale bars: 50 µm.

Freshly isolated mammary gland fragments contain native ECM, stromal cells and undefined additional components that can influence MGO development. To remove such confounding factors, we purified single mammary epithelial cells (MECs) and find that also purified MECs seeded in PIC hydrogels (2 mg mL^−1^ with 63 × 10^−6^ m RGD) develop into cystic MGOs (**Figure** **3**a). Analogously to what we observed in the long‐term cultures, around half of these MGOs have K14‐positive basal cells on the outer layer and K8‐positive luminal cells on the inner layer, while in the remaining MGOs contained K8‐positive luminal cells only (Figure 3b).

Basal cells are known to be bi‐potent and can generate either basal or luminal cells in a normal mammary gland.^[^ ^24^ ^]^ As such, K8 and K14‐positive MGOs are expected to originate from the basal population. In contrast, luminal cells commonly do not differentiate into basal cells, which gives rise to only K8 positive organoids,^[^ ^24^ ^]^ which explains our earlier observation that some organoids generated from single cells are both K14 and K8 positive while others are only K8 positive. To confirm our hypothesis, we separated the purified MECs into basal or luminal populations by fluorescence‐activated cell sorting (FACS) sorting^[^ ^46^ ^]^ (Figure 3a). Indeed, the purified basal cell population developed into MGOs, which contained K14‐positive basal cells outside and K8‐positive luminal cells when cultured in PIC hydrogels or in Matrigel (Figure 3b,c). In contrast, the purified luminal cell population cultured in either gel shows cystic MGOs that are K8‐positive and are smaller in size. Interestingly, MGOs derived from the unsorted cells as well as the purified basal population, but not from the purified luminal cells, fully retained the capacity to branch when reseeded in Matrigel (Figure 3c–e). In short, also purified MECs cultured in PIC hydrogels are able to form into MGOs.

### 2.5 PIC Hydrogels as Tunable Organoid Culture Matrix

The ECM has a crucial instructive role in mammary gland development and differentiation. Important parameters that determine how the ECM affects cell behavior include its stiffness and the relative abundance of different ECM proteins.^[^ ^47^, ^48^ ^]^ Cells sense and respond to these differences through integrins, which bind peptide motifs such as the RGD motif found in many ECM proteins.^[^ ^42^ ^]^ One of the key advantages of using synthetic polymers as matrix for cell culture application is that such parameters (stiffness, stress relaxation, critical stress, peptide density, etc.) can be readily modified, sometimes independently.^[^ ^30^, ^31^ ^]^ To investigate how the PIC hydrogel stiffness and the RGD ligand density affect the basal and luminal cell populations during MGO formation, we seeded mammary gland fragments in four different PIC hydrogels.

The 0.2 wt% PIC hydrogel that we described in the earlier sections (labeled PIC‐1) contains 63 × 10^−6^ m RGD and has a stiffness of 0.32 kPa. In PIC‐2, PIC‐3, and PIC‐4, we varied the RGD density and stiffness independently (**Table** **1** and Figure S6, Supporting Information).

**Table 1:** PIC hydrogels and physical properties of the gels. Note that P1 was prepared by post‐modification of P1‐azide (viscosity‐average molecular weight *M*_v_ = 502 kg mol^−1^), and that both have nearly the same polymer characteristics

Hydrogel	Polymer	Concentration *c* [mg mL^−1^]	Concentration RGD [µm]	*G′_T_*_= 37 °C_ [kPa]
PIC‐1	P1	2	63	0.32
PIC‐2	P1‐azide	8	0	2.4
PIC‐3	P1 + P1‐azide (1:3)	8	63	2.3
PIC‐4	P1	8	252	2.3

**Figure 4:**
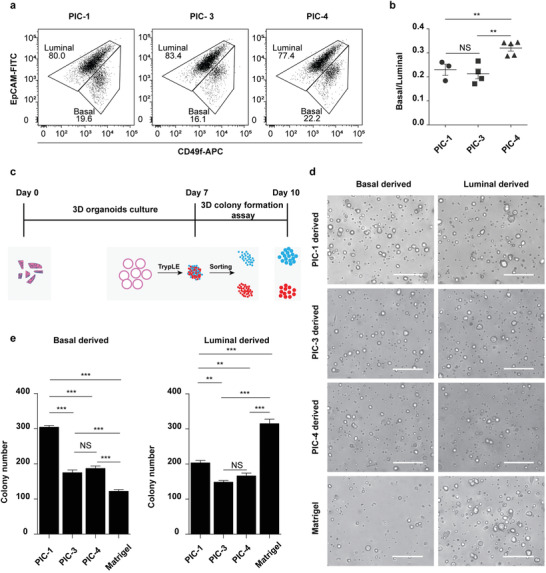
PIC hydrogels provide a tunable system to investigate specific MGO characteristics. a) Representative FACS plots of CD49f (basal) and EpCAM (luminal) stained cystic MGOs generated in different PIC hydrogels. One set of representative FACS plots out of three independent experiments is shown. b) Quantification of basal to luminal population ratio of cystic MGOs generated in different PIC hydrogels from FACS analysis. c) Schematic description of the 3D colony formation assay of mammary epithelium of cystic MGOs generated in different PIC hydrogels. d) Representative bright‐field images of 3D colonies generated from PIC hydrogels or Matrigel‐derived basal or luminal population. Two independent experiments were performed and data was presented for one of the experiments. The results of the other experiment are shown in Figure S8 (Supporting Information). Scale bar, 250 µm. e) Quantification of basal/luminal population‐derived 3D colony numbers in PIC hydrogels or Matrigel. Only colonies with a diameter above 20 µm were counted. NS, not significant. ** *P* < 0.01, *** *P* < 0.001, using one‐way ANOVA followed by Tukey's multiple comparisons test.

Embedding fresh mammary gland fragments in these different hydrogels yielded cystic MGOs in all samples besides PIC‐2 that does not contain RGD (Figure S7a, Supporting Information), which then was left out of further analyses. These data thus show that the presence of RGD in PIC hydrogels is indispensable for proliferation and polarized organization of MGOs. Viability of the cells embedded in the RGD‐functionalized PIC hydrogels was measured by FACS and was similar to PIC‐1 and to the Matrigel cultures (Figure S7b, Supporting Information). After seven days, the cystic MGOs were harvested and the basal and luminal cell populations were quantified by FACS (**Figure** **4**a). We observed no significant differences in the basal to luminal cell ratio between softer *G*′ = 0.3 kPa (PIC‐1) and stiffer *G*′ = 2.3 kPa (PIC‐3) hydrogels (Figure 4b). Interestingly, a significantly higher fraction of basal cells was detected in PIC hydrogels containing an increased RGD density (63 × 10^−6^ m in PIC‐3 to 252 × 10^−6^ m in PIC‐4) (Figure 4b). These results suggest that an increased engagement of RGD‐interacting integrins leads to signaling that biases differentiation toward a basal cell lineage and that integrin activation by RGD‐functionalized PIC hydrogels in the concentration range used here is a limiting factor of basal cell development in cystic MGOs.

The mammary epithelium contains terminally differentiated cells, but is also rich in progenitors. The latter are of particular interest in the context of breast cancer as they are likely targets of malignant transformation in the mammary gland. We quantified the presence of progenitor cells using a traditional 3D colony formation assay where the number of discrete 3D colonies is a good measure for the original progenitor concentration.

Mammary gland fragments were embedded in PIC‐1, PIC‐3, PIC‐4 and in Matrigel as a control. After seven days, the basal and luminal cell population were FACS sorted and reseeded in Matrigel for the 3D colony formation assay (Figure 4c). After three days in culture, the basal cells that were grown in the softer hydrogels (PIC‐1, *G*′ = 0.3 kPa) formed significantly more colonies than basal cells in the stiffer hydrogels (PIC‐3, *G*′ = 2.3 kPa); an effect that was also seen with luminal cells albeit less pronounced (Figure 4d,e). In contrast, a change in the RGD density (63 × 10^−6^ m for PIC‐3 to 252 × 10^−6^ m for PIC‐4) did not change the progenitor concentrations in the basal and luminal cell populations (Figure 4d,e). Strikingly, basal cells seeded in any of the PIC hydrogels showed a higher progenitor density when compared to those in Matrigel, whilst luminal cells had the highest density in Matrigel. Altogether, our data shows that in PIC hydrogels, the RGD density affects the MGO cell composition (basal vs luminal cell density) and not their colony formation efficiency, whereas the stiffness does not affect the composition, but does change the colony formation efficiency.

## 3 Discussion

The mouse mammary gland in vivo is surrounded by a basement membrane containing laminin, collagen IV, nitrogen and heparin sulfate proteoglycans. These different matrix compounds induce different morphologies and functionalities of the resulting organoids.^[^ ^47^ ^]^ For example, MGOs embedded in Matrigel containing collagen IV and laminin‐1 express a higher level of genes encoding milk proteins, but a collagen‐I‐rich ECM favors tubular growth under the right hormonal stimulation.^[^ ^49^ ^]^ One of the major challenges is to understand how the ECM environment controls organoid formation and morphogenesis. We here describe the first well‐defined synthetic hydrogel with minimal components that supports (cystic) MGO formation.

Previously, a poly(ethylene glycol)‐glycosaminoglycan (PEG‐GAG)‐based synthetic hydrogel was successfully used for acinar growth and apical‐basal polarization of breast epithelial cells.^[^ ^50^ ^]^ This study used the MCF10A cell line, that shows no signs of terminal differentiation and displays characteristics of luminal, but not basal cells, and has not been shown to be capable of branching in either natural or artificial matrices.^[^ ^51^ ^]^ In our work, we use primary murine mammary epithelium to establish mammary organoids. Mammary gland fragments as well as purified single basal cells self‐organize into bi‐layered MGOs that encompass an inner luminal layer and an outer basal cell layer. Our PIC hydrogels thus allow to closely mimic the in vivo self‐organizing process that involves different cell populations (e.g., basal and luminal cells). MGOs embedded in PIC hydrogels can be cultured for a prolonged period of time without losing their branching capacity. We show that variations in the hydrogel induce changes in MGO composition: the RGD concentration determines the basal/luminal cell ratio and the gel stiffness regulates the progenitor density, which is always higher than in the Matrigel control.

### 3.1 MGO Formation

Regulation of cell polarization and lumen formation has been extensively studied in 3D cell models and in vivo, and depends on the interaction of mammary epithelial cells with a basement membrane, and downstream *β*1‐integrin dependent cell‐ECM signaling.^[^ ^52^ ^]^ Indeed, we found that organoid formation is broadly supported by PIC hydrogels as long as they are decorated with the RGD peptide, which is known to bind several *β*1‐containing integrins.^[^ ^42^ ^]^ The requirement of cell‐binding interactions for organoid growth is in line with earlier work on murine intestinal organoids cultured in fibrin‐based^[^ ^53^ ^]^ and in synthetic PEG hydrogels.^[^ ^31^ ^]^ Additionally, we experimentally find that the concentration of RGD is an important factor that determines the basal/luminal cell ratio; a higher RGD concentration enhances the proliferation of the basal cell population. The data is consistent with earlier work that found that increased fibronectin–also containing the RGD motif–levels resulted in higher proliferation of mammary epithelial cells.^[^ ^7^ ^]^

### 3.2 Branching Morphogenesis

Branching is a fundamental and complex aspect of mammary gland development. In vivo, branching is relatively slow (weeks), much slower than in many in vitro cultures (days).^[^ ^25^ ^]^ The exact physical and chemical mechanisms that drive branching remain unclear and, so far, no single factor has been defined for mammary gland branching.^[^ ^25^, ^54^ ^]^ In fact, many different factors contribute, including the ECM, hormones, growth factors and cells present in the extracellular environment.^[^ ^25^, ^55^ ^]^ For instance, recent work showed that organoid branching in Matrigel is stimulated by FGF2^[^ ^56^ ^]^ but when we added FGF2 to the PIC MGO cultures, it did not induce branching (not shown). As for the matrix, local degradation by matrix metalloproteases stimulates outward budding of intestinal organoids in PEG gels,^[^ ^31^ ^]^ whereas local densification of ECM can inhibit this process.^[^ ^51^ ^]^ The fibrous network of PIC hydrogels is not biodegradable, although proliferation and morphogenesis seem unhindered by the presence of the network, which is physically remodeled.^[^ ^57^ ^]^ Earlier, Zimoch et al.^[^ ^39^ ^]^ used PIC hydrogels to elegantly show that endothelial cells can form into their native structures as branching tubes in a system that involves a single cell type in short‐term experiments. MGO formation, however, is a more complex developmental process involving different primary cell types that all need to be spatially reorganized in order to be able to form polarized organoids. PIC hydrogels support this complex process and maintain the branching capacity of MGOs over a prolonged period of culturing, although we do not observe branching in the PIC hydrogels themselves. Nowak et al. found that in PEG‐GAG‐based hydrogels, matrix metalloproteinase‐cleavable crosslinkers and heparin could promote invasion of epithelial cells into the matrix, but did not allow for branching of polarized tubular structures, as is observed for MGOs in Matrigel.^[^ ^50^ ^]^ Likely, the addition of MMPs may act directly on the spherical colonies, described by the authors, and thus introduces extra variables. In contrast, the PIC hydrogels we describe are physically crosslinked and form a matrix with RGD as sole cell adhesion ligand. Therefore, the PIC hydrogels provide a more controllable and predictable model system. Capturing the organoids in an early developmental state makes the RGD‐functionalized synthetic materials particularly attractive as minimal models to decipher which factors or combination of factors are required to induce and control MGO branching, a task that is very difficult to realize in a signal‐rich matrix such as Matrigel.^[^ ^51^ ^]^

### 3.3 Physical Cues from the Matrix

Artificial matrices are well‐suited to maintain stem cells in their undifferentiated state.^[^ ^58^, ^59^, ^60^ ^]^ While the contribution of the mechanical properties of these matrices is widely recognized, the effects still are ambiguous. For instance, Gilbert et al.^[^ ^61^ ^]^ reported that muscle stem cells (MuSCs) cultured on soft hydrogels (12 kPa, mimicking the elasticity of muscle tissue) self‐renew in vitro and contribute extensively to muscle regeneration, in contrast to MuSCs cultured on rigid tissue culture plastic. Oppositely, Musah et al.^[^ ^62^ ^]^ found that only stiff substrates are able to maintain human embryonic stem cell proliferation and pluripotency. We find that MGOs cultured in softer hydrogels contain more progenitors, which contrasts the results of the intestinal stem cells in PEG gels,^[^ ^31^ ^]^ which showed low expansion in soft hydrogels (0.3 kPa, 1 × 10^−3^ m RGD) compared to stiffer hydrogels (1.3 kPa, 1 × 10^−3^ m RGD). Recent developments, however show that the mechanical properties are much more than just the elasticity (storage modulus) of the hydrogels; stress relaxation,^[^ ^63^, ^64^ ^]^ stiffening induced by contractile cells^[^ ^37^, ^38^ ^]^ and matrix degradation^[^ ^31^ ^]^ (rates) all affect the cell response. Clearly further studies are necessary to delineate the effects of the matrix mechanics on the expansion capacity of mammary progenitor cells, and whether additional factors, including biomolecules such as RGD or others, play a role. On a more general level, synthetic matrices are well suited for these studies, because they lack the high and poorly controlled signal density of natural basement membrane matrices, and because minimal changes in the matrix properties can be used to drive cell behavior.

## 4 Perspective

In this work, we studied minimal PICs hydrogels decorated with the well‐known RGD peptide. Compared to Matrigel that has a shear modulus of *G*′ ≈ 0.05 kPa, PIC hydrogels have a tunable shear modulus (*G*′ ≈ 0.1–4 kPa) that better overlaps with the stiffness of mammary adipose tissue (*G*′ ≈ 1 kPa). The PIC conjugation strategy allows for the introduction of a large number of peptides or larger biomolecules and its thermosensitivity makes subsequent analyses relatively facile: by simply cooling the gel and centrifugation, the MGOs can be harvested. In absence of abundant signaling factors, relatively small variations in the PIC hydrogels give rise to large changes on the cellular composition and organization of the MGOs. This minimal nature of the matrix makes PIC hydrogels ideal platforms to study parameters affecting key processes such as polarization and branching in organoid systems and tissue development. In addition, the combination of a biomimetic synthetic matrix and excellent biocompatibility offers an ultimate prospect of in vivo applications.

## 5 Experimental Section

##### Synthesis of PIC‐Azide and PIC‐RGD

The synthetic hydrogels are based on oligo(ethylene glycol)‐grafted polyisocyanides. The preparation of azide‐functionalized PICs and RGD‐functionalized PICs as summarized in Figure S1 (Supporting Information), follows previous reported procedures.^[^ ^33^, ^37^, ^38^ ^]^ In short, the co‐polymerization of tri(ethylene glycol)‐grafted isocyano‐(d)‐alanyl‐(l)‐alanine monomer 1 and the azide‐appended monomer 2 (molar ratio of 1:2 = 99:1) was catalyzed by nickel perchlorate (total monomer:catalyst ratio 3000:1 (3K, P1‐azide) (or with different ratios for the preliminary experiments: 1000:1 (1K) and 5000:1 (5K). Viscometry analysis^[^ ^34^ ^]^ yielded viscosity averaged molecular weights *M*_v_ = 502 kg mol^−1^ (3K), 322 kg mol^−1^ (1K), and 530 kg mol^−1^ (5K). Raw experimental data is given in Figure S9 (Supporting Information). The azide groups on the polymers were reacted with acetylene‐equipped cell‐binding peptides (RGD) through the highly efficient strain‐promoted azide‐alkyne cycloaddition reaction following literature procedures.^[^ ^37^ ^]^ To form a gel, the desired amount of sterile HBSS (Sigma Aldrich, Cat. #H4891, prepared by dissolving Hank's Balanced Salts, 1 g in 1 L sterile H_2_O) was added to the solid, sterilized (UV, 5 min) polymer. After overnight soaking at 4 °C, the mixture was shaken vigorously for a few seconds and a transparent solution was formed. When the cold transparent solution is heated above its gelation temperature, it immediately forms a soft, (visco)elastic gel. During the whole study, a large batch of PIC hydrogels in HBSS was prepared, aliquoted and frozen at −20 °C until use. Before each experiment, the frozen hydrogels were firstly placed on ice to thaw.

##### Rheology Measurements

Rheological measurements were performed on a stress‐controlled rheometer (Discovery HR‐2, TA Instruments) using a steel parallel plate geometry with a diameter of 40 mm and a gap of 500 µm. All samples were loaded on the rheometer plate as cold solutions (5 °C). To determine the storage modulus *G*′, the sample was subjected to an oscillatory deformation of amplitude *γ* = 0.04 at a frequency *ω* = 1.0 Hz. The gelation temperature (determined from a heating ramp with rate 1.0 °C min^−1^) was taken as the onset of the increase in storage modulus *G*′. Viscometry was used to measure the viscosity average molecular weight *M*_v_ and to estimate the polymer length (*L*) as previously described.^[^ ^36^ ^]^

##### Mice

All mice used here were C57BL/6 (8–12 weeks old) and housed at the Animal Research Facility (ARF) of the Radboud University Medical Center (Charles River). All animal experiments were documented and approved by the Animal Experimental Committee of the Radboud University Medical Center and were performed in accordance with regulatory standards of the Animal Experimental Committee.

##### Mammary Gland Fragments Isolation

Mouse mammary gland fragments were isolated according to previous protocol.^[^ ^23^ ^]^ After the mammary glands (#3, #4, and #5) were detached from the mouse, they were chopped into small pieces with scalpels. The minced glands were incubated in 2 µg mL^−1^ collagenase A (Sigma‐Aldrich, Cat. # C5138) in DMEM/F12 medium (DMEM/F12 (Gibco, Cat. #11 330 057) with 5 µg mL^−1^ Insulin (Sigma, Cat. #I1882), 1% Antibiotic‐Antimycotic (Gibco, Cat. #15 240 062), and 5% fetal bovine serum (Merck, Cat. #F7524)) for 30–40 min at 37 °C with 150 rpm shaking. After removal of the collagenase A solution, the fragments were washed with DMEM/F12 medium. The supernatant was discarded and the sediment was treated with 40 U mL^−1^ DNase I (Roche, Cat. #11 284 932 001) in DMEM/F12 medium for 3–5 min at room temperature by gently inverting by hand to break up the clusters and detach the fragments from adhered single cells. The supernatant was removed and the pellet was resuspended in DMEM/F12 medium and centrifuged at 1500 rpm to remove single cells and tissue debris. The final pellet was subsequently dissociated into single cells or directly embedded in PIC hydrogels or Matrigel (Corning, Cat. #354 230). When required, a single‐cell suspension was obtained by sequential dissociation of the fragments by incubating in TrypLE (Gibco, Cat. #12605‐010) for 15–20 min at 37 °C.

##### Mammary Gland Organoids Culture

Freshly isolated mouse mammary gland fragments (50–100 pieces per 50 µL gel) or dissociated single mammary epithelial cells (10 000–30 000 cells per 50 µL gel) were mixed with 50 µL Matrigel or PIC hydrogels and seeded at the bottom of 24‐well plates. After hydrogels had gelated in the incubator at 37 °C (30 min for Matrigel, 1 h for PIC hydrogels), 700 µL culture medium (Table S1, Supporting Information) was added per well and organoids were cultured in a 37 °C humidified atmosphere under 5% CO_2_. In general, culture medium was refreshed every three days.

##### Mammary Gland Organoids Branching Ability Assay

After formation in Matrigel or PIC hydrogels, MGOs were collected by simply cooling to 10 °C for 10 min and washing with cold PBS twice. Then, the MGOs were mixed with fresh Matrigel and seeded in 24‐well plates. After gelation, 3D organoid culture medium for fragments was added. MGO changes in morphology were recorded on day three by a Zeiss Vert. A1 microscope.

##### Mammary Gland Organoids Maintenance In Vitro

MGOs were maintained in PIC hydrogels for two months by passaging them every week. Briefly, every seven days, organoids were removed from the PIC hydrogels by simply cooling at 10 °C for 10 min and washed with cold PBS twice, and treated with TrypLE for 15–20 min at 37 °C before being transferred to fresh PIC hydrogels. After seeding in 24‐well plates, cells were overlaid with 3D organoid culture medium (Table S1, Supporting Information).

##### Prostate Organoid Culture

Prostate organoid culture was performed as described previously.^[^ ^65^ ^]^ Briefly, minced prostate tissue from 8 to 12 weeks old male mice was digested in 5 mg mL^−1^ collagenase type II solution (Life Technologies, cat. no. 17101‐015) with 10 × 10^−6^ m Y‐27632 dihydrochloride (Abmole Bioscience, cat. no. M1817) on a shaking platform at 37 °C for 1–1.5 h. After aspirating, the collagenase type II solution, a single prostate epithelial cell suspension was generated by incubating samples in TrypLE with 10 × 10^−6^ m Y‐27632 dihydrochloride at 37 °C for 15 min. 20 000 prostate epithelial cells were embedded in a 40 µL Matrigel or PIC hydrogel drop and seeded in the middle of one well of a 24‐well tissue culture plate. After gelation of the Matrigel or PIC hydrogel, 500 µL prewarmed 3D prostate organoid culture medium (Table S1, Supporting Information) was added and organoids were cultured in an incubator (5% CO_2_, 37 °C). Culture medium was refreshed every 2–3 days.

##### Flow Cytometry and Fluorescence‐Activated Cell Sorting

After freshly isolated mouse mammary gland fragments were cultured in PIC hydrogels or Matrigel for seven days, the generated MGOs were collected as described above. After washing with cold PBS twice, MGOs were digested in TrypLE for 15–20 min at 37 °C to obtain single‐cell suspensions of mammary epithelium. For flow cytometry and sorting, cells were stained with CD31‐PE/cy7 (Antibodychain, Cat. #1 112 040), CD45.2‐BV510 (Antibodychain, Cat. #1 149 185), TER119‐PE (eBioscience, Cat. #12‐5921‐81), CD45.2‐PerCP (BD, Cat. #552 950), EpCAM‐FITC (eBioscience, Cat. #11‐5791‐82), and CD49f‐APC (eBioscience, Cat. #17‐0495‐80). Flow cytometry analysis was conducted on BD FACS Verse and FACS sorting was performed on a FACS ARIA III. Fixable Viability Dyes (FVD) eFluor 450 was added to distinguish dead and live cells. Data were analyzed using FlowJo V10 software and FACS data are presented in dot plots with equal number of events. The gating strategy for the mammary epithelium is shown in Figure S10 (Supporting Information).

##### 3D Colony Formation Assay

MGOs generated from fresh mammary gland fragments within Matrigel or PIC hydrogels were collected and digested into a single‐cell suspension. After the single‐cell suspension was sorted based on the description above, freshly sorted basal or luminal cells were resuspended at a density of 30 000 cells mL^−1^ in cold 100% Matrigel (Corning, Cat. #354 230) and overlaid with 3D colony formation medium (Table S1, Supporting Information) following 30 min polymerization at 37 °C. The culture medium was refreshed every 48 h. To quantify the colony numbers, phase contrast z‐stacks spanning the entire thickness of the gel were collected at two different positions within each gel on day three after seeding. Different layers were aligned and blended into one layer with Photoshop software to quantify all the colonies. The results are representative of two independent experiments performed with three gel samples per experimental group. In each independent experiment, five to six mice were pooled.

##### Immunofluorescence Analysis

For immunofluorescence analysis, MGOs generated within Matrigel or PIC‐RGD hydrogels were fixed with 4% paraformaldehyde (PFA) in PBS at room temperature for 1 h. After removal of the supernatant, the pellet was incubated in 1.5% eosin at room temperature for 5 min. Following further washing with PBS, the pellet was resuspended in 2.25% agar solution of 80–90 °C. The hot agar solution with organoids were centrifuged (7200 rpm, 2 min). The solidified agar solution with organoids was embedded in paraffin. Sections of 2 µm thickness were cut using a microtome. Individual sections were mounted onto superfrost slides and dried overnight at 37 °C. After deparaffination with Histochoice (VWR, Cat. #H103‐4L) twice for 10 min each, samples were rehydrated with 100% (2  × 1 min), 96% (2 × 1 min), and 70% (1 × 1 min) ethanol, followed by washes with tap water (2 × 1 min). The slides were then heated for 15 min in citrate buffer (pH 6.0) (Dako, Cat. #S1699) in a microwave oven for antigen retrieval. After 1 h of cooling down, the samples were then blocked in 2% BSA/PBS at room temperature for 1 h. Primary antibody incubation was performed in 1% BSA/PBS at room temperature for 2 h. The following primary antibodies were used: rabbit anti‐cytokeratin 8 (K8, Abcam, Cat. #Ab53280, 1:200) and mouse anti‐cytokeratin 14 (K14, Abcam, Cat. #Ab7800, 1:200). Secondary antibody incubation was in 1% BSA/PBS at room temperature for 1 h, followed by three PBS washes. Alexa 488 (Invitrogen, Cat. #A21206, 1:400) or 568 (Invitrogen, Cat. #A11031, 1:400) conjugated secondary antibodies were used. The following primary antibodies were used for prostate organoids: mouse p63 (Abcam, Cat. #Ab735, 1:200), mouse High Molecular Weight (HMW, clone 34BE12, Abeomics, Cat. #34BE12) and K8 (Abcam, Cat. #Ab53280, 1:200). Secondary antibody incubation was in 1% BSA/PBS at room temperature for 1 h, followed by three PBS washes. Alexa 488 (Invitrogen, Cat. #A21202, 1:400) or 594 (Invitrogen, Cat. #A21207, 1:400) conjugated secondary antibodies were used. All immunofluorescence experiments were performed with negative controls where relevant isotype was added (Mouse isotype: Biolegend, Cat. #400 102, 1:500; rabbit isotype: Cell Signaling Technology, Cat. #3900, 1:15 000). The samples were then incubated with DAPI (5 µg mL^−1^) at room temperature for 10 min, followed by three PBS washes. The slides were mounted in anti‐fade medium (Fluoromount W for microscopy, Serva), and images were acquired using a Leica DM6000 microscope (Leica). Acquired images were processed by Fiji.

##### Statistical Analysis

Statistically significant differences were assessed by using one‐way ANOVA followed by Tukey's multiple comparisons test in the GraphPad Prism 5.0 software. *P* values of statistical significance are represented as * *P* < 0.05, ** *P* < 0.01, *** *P* < 0.001, NS, not significant.

## Conflict of Interest

The authors declare no conflict of interest.

## Supporting information

Supporting Information
