# Supplementary material for: Polyisocyanide Hydrogels as a Tunable Platform for Mammary Gland Organoid Formation
Source: Adv Sci (Weinh). 2020 Jul 26;7(18):2001797. doi: 10.1002/advs.202001797 (PMC7509700; doi:10.1002/advs.202001797)
Supplement: Supplementary file 1 — Supporting Information [file ADVS-7-2001797-s001.pdf]

Copyright WILEY-VCH Verlag GmbH & Co. KGaA, 69469 Weinheim, Germany, 2020.

ADVANCED SCIENCE Open Access

Supporting Information

for *Adv. Sci.,* DOI: 10.1002/advs.202001797

Polyisocyanide Hydrogels as a Tunable Platform for Mammary Gland Organoid Formation

Ying Zhang, Chunling Tang, Paul N. Span, Alan E. Rowan, Tilly W. Aalders, Jack A. Schalken, Gosse J. Adema, Paul H. J. Kouwer,* Mirjam M. P. Zegers, and Marleen Ansems*

Supplementary Information

Table S1. Overview composition of culture media used in the study.

	3D Organoids medium- mammary gland fragments	3D Organoids medium- (single) mammary epithelial cells	3D colony formation medium	3D Organoids medium- prostate epithelial cells	Company
AdDMEM/F12		+		+	Gibco Cat. #12634034
DMEM/F12	+		+		Gibco Cat. #11330057
HEPES		+ 10 mM			Thermo fisher Cat. #15630-056
Antibiotic-Antimycotic	+1X	+1X	+1X		Gibco Cat. #15240062
Ultra-glutamine		+ 2 mM			Lonza Cat. #BE17-605E/U1
Insulin		+5 µg ml^{-1}			Sigma Aldrich Cat. #I1882-100MG
Hydrocortisone		+100 ng ml^{-1}			Sigma Aldrich Cat. #H0888-1G
FGF2	+50 ng ml^{-1}	+5 ng ml^{-1}			Sigma Cat. #F0291-25UG
EGF		+50 ng ml^{-1}	+50 ng ml^{-1}	+50 ng ml^{-1}	Peprotech Cat. #AF-100-15
ITS	+1%		+1%		Gibco Cat. #51500
FGF10		+10 ng ml^{-1}			Peprotech Cat. #100-26
B27		+1X		+1X	Thermo fisher Cat. #17504044
N-Acetyl-L-cysteine		+1.25 µM		+1.25 µM	Sigma Aldrich Cat. #A7250-5G
Y-27632		+5 µM			BioConnect Cat. #S1049_2mg
A83-01				+0.2 µM	Tocris Bioscience Cat. #2939
Noggin				+100 ng ml^{-1}	Peprotech Cat. #120-10C
R-spondin 1				+500 ng ml^{-1}	R&D Systems Cat. #4645-RS-025
Dihydrotestosterone				+1nM	Selleckchem Cat. #S4757

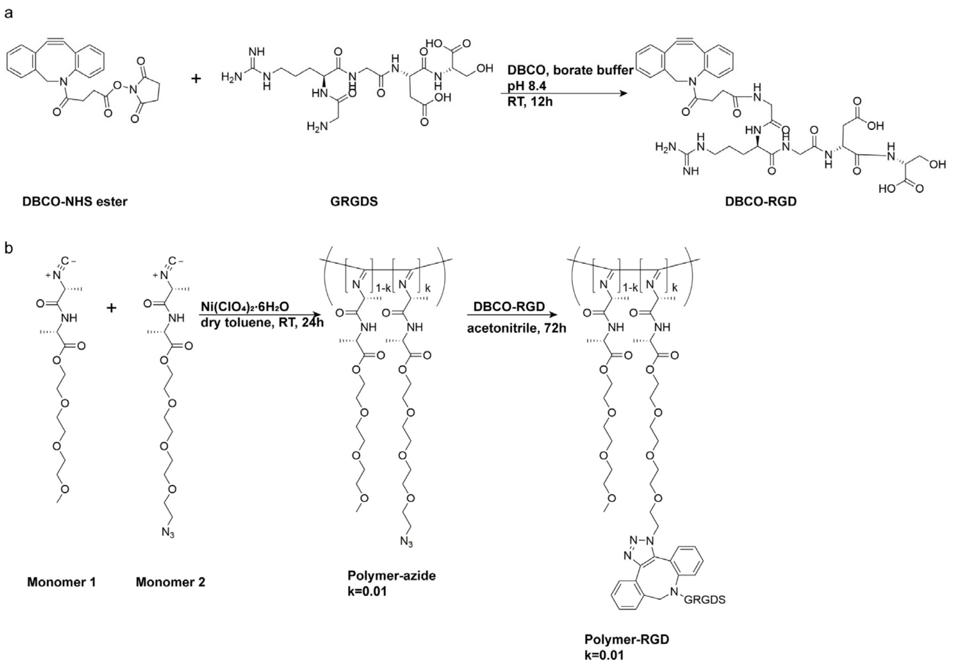

Figure S1. a Synthesis of intermediate DBCO-GRGDS. **b** Polymerization of PIC-azide and subsequent functionalization through click chemistry to generate PIC-1(0.2 wt-%, 63 µM RGD, *G′* = 0.32 kPa,). The distribution of RGD groups is random with an average of every 14-18 nm along the polymer chain. RT = room temperature.

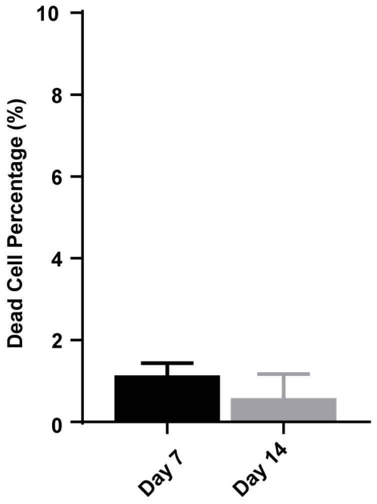

Figure S2. Bar graph shows the percentage of dead cells within MGOs formed in PIC hydrogel (0.2 wt-%, 63 µM RGD, *G′* = 0.32 kPa,) on day 7 and day 14 respectively during culture. Dead cells were quantified by counting the amount of cell with defected nuclear.

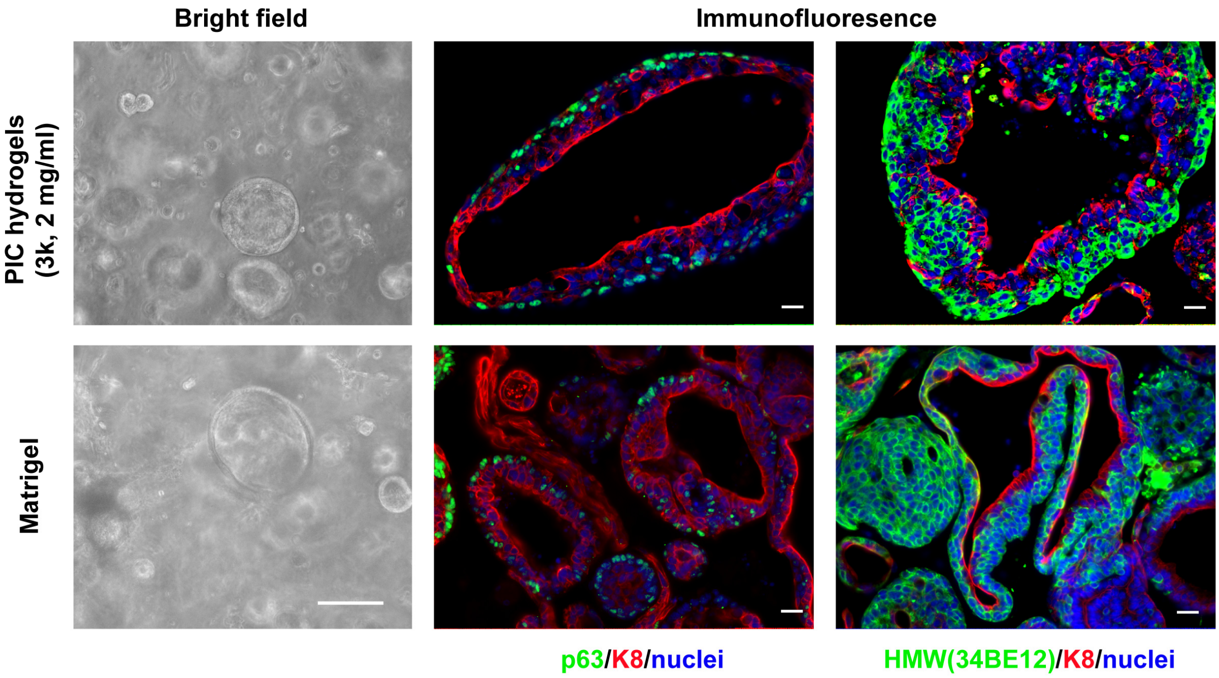

Figure S3. Representative bright-field and immunofluorescence images of mouse prostate organoids formed in PIC hydrogel (0.2 wt-%, 63 µM RGD, *G′* = 0.32 kPa) hydrogels and Matrigel on day 7, respectively. Scale bars: 250 µm (bright-field images), 20 µm (immunofluorescence images). Basal cells are labeled with p63 (green) or HMW (34BE12) (green), Luminal cells are labeled with K8 (red), and nuclei are stained with DAPI (blue).

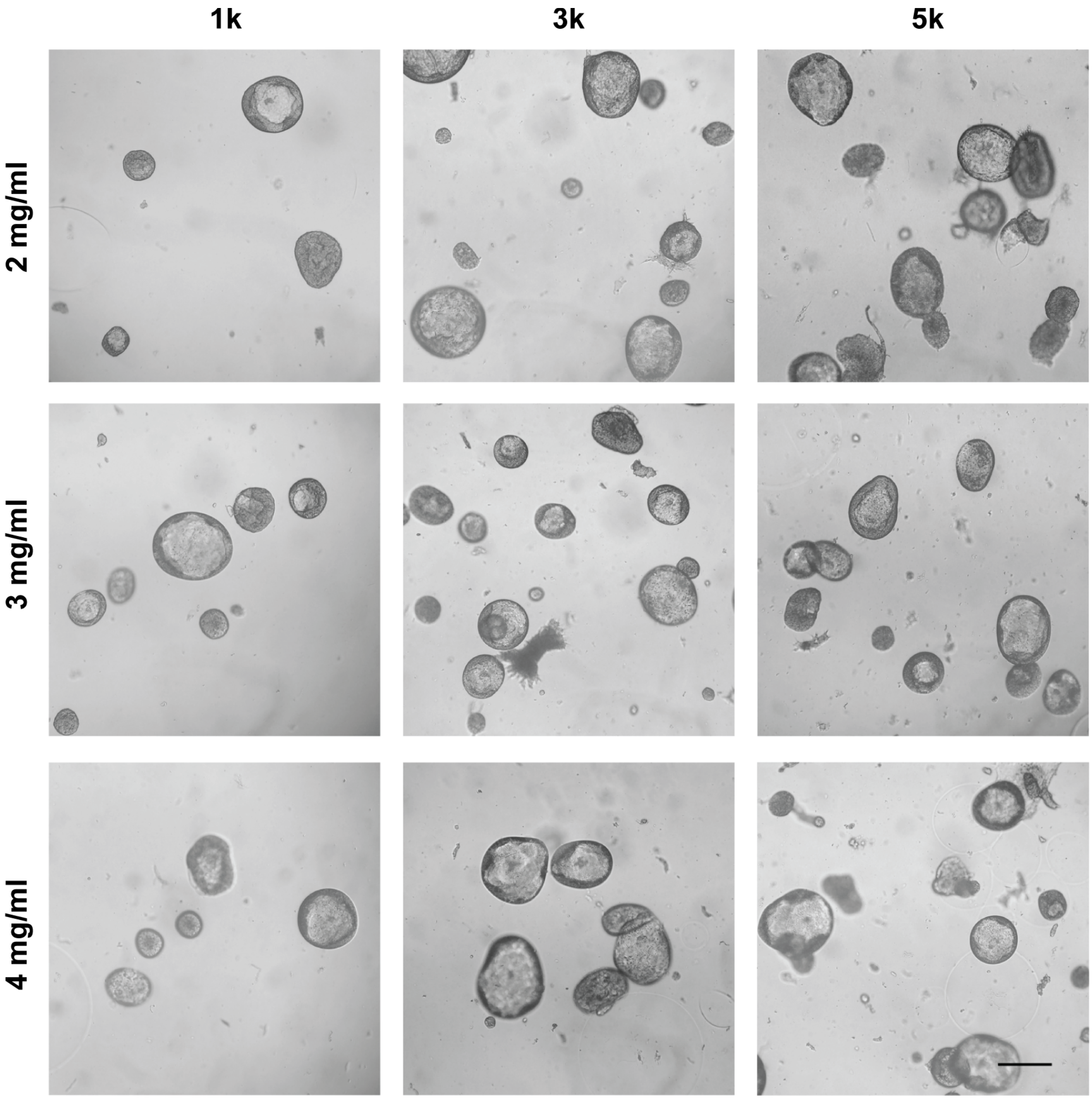

Figure S4. Representative images of MGOs formed in PIC hydrogels with different concentrations and molecular weight on day 7 during culture. Mv = 322 kg mol^{-1} (1K), Mv = 502 kg mol^{-1} (3K), Mv = 530 kg mol^{-1} (5K), Scale bar: 100 µm.

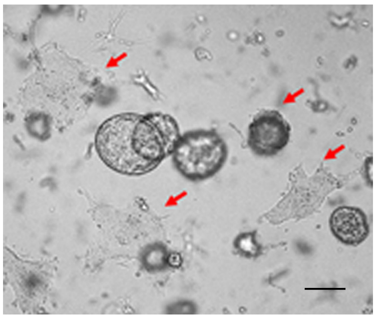

Figure S5. Representative image of MGOs formed in PIC hydrogel with low stiffness (0.1 wt-%, 63 μM RGD, *G′* = 20.5 Pa) on day 7 during culture. Arrows indicate mammary organoids are attached to the bottom of the plate. Scale bar: 100 μm.

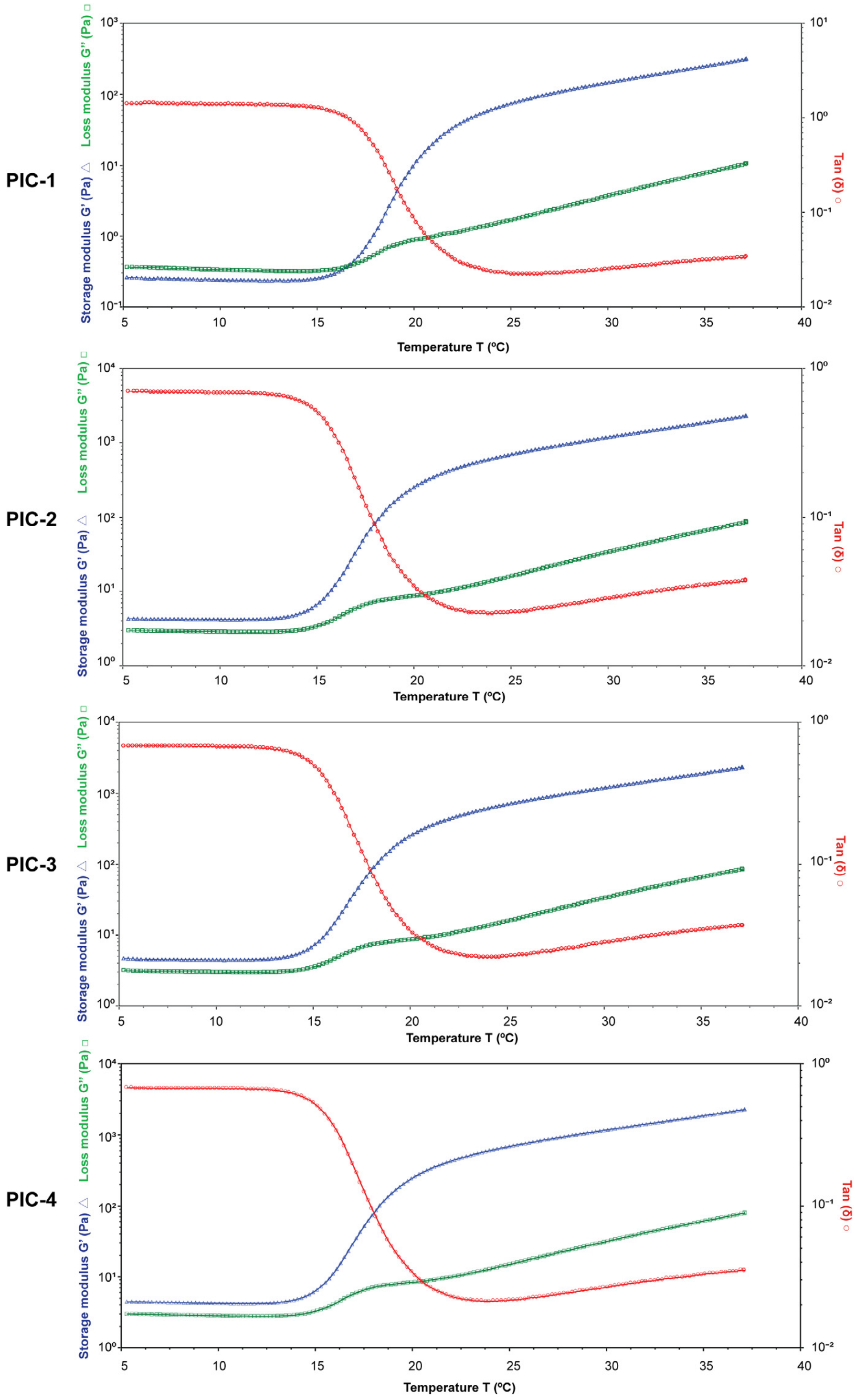

Figure S6. Rheology data of PIC-1(0.2 wt-%, 63 μM RGD, *G′* = 0.32 kPa), PIC-2 (0.8 wt-%, 0 μM RGD, *G′* = 2.4 kPa), PIC-3 (0.8 wt-%, 63 μM RGD, *G′* = 2.3 kPa) and PIC-4 (0.8 wt-%, 252 μM RGD, *G′* = 2.3 kPa) (HBSS buffer, 37 °C). We define the final point at 37°C as the stiffness of hydrogels.

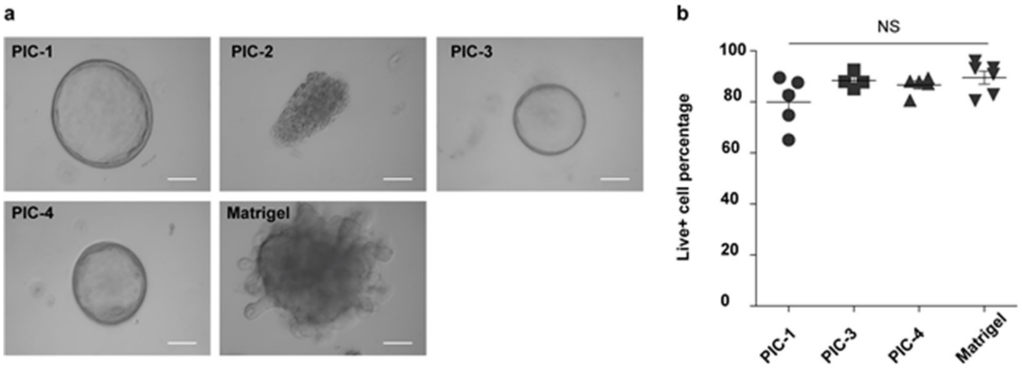

Figure S7. PIC hydrogels stiffness and RGD effect on MGOs formation and viability. PIC-1(0.2 wt-%, 63 μM RGD, G' = 0.32 kPa), PIC-2 (0.8 wt-%, 0 μM RGD, G' = 2.4 kPa), PIC-3 (0.8 wt-%, 63 μM RGD, G' = 2.3 kPa) and PIC-4 (0.8 wt-%, 252 μM RGD, G' = 2.3 kPa). **a** Representative bright-field and fluorescence microscopy images of MGOs cultured in PIC hydrogels with different stiffnesses and RGD densities or Matrigel on day 7. Scale bar: 50 μM. **b** Percentage of live cells from MGOs derived from PIC-RGD hydrogels of different stiffnesses and RGD densities or Matrigel, which was quantified from FACS after mammary gland fragments being encapsulated in gels for 7 days in five independent experiments. Fixable Viability Dyes (FVD) eFluorTM 450 were utilized to stain dead cells. NS = not significant, one-way ANOVA followed by Tukey's multiple comparisons test.

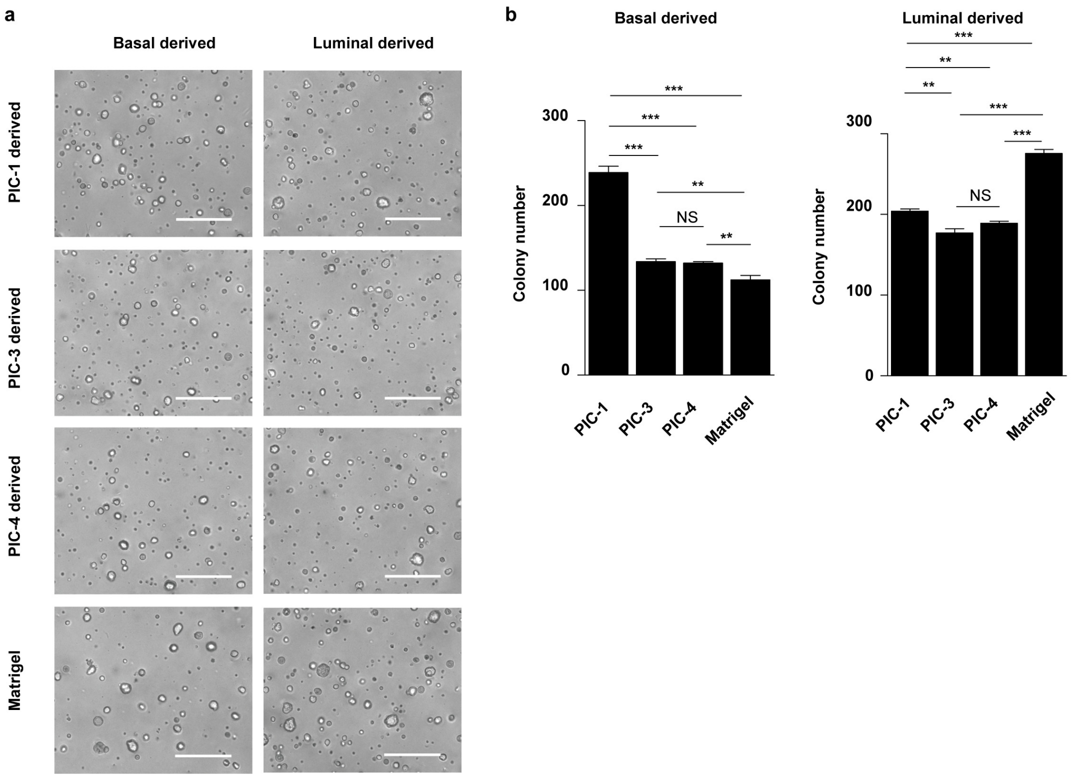

Figure S8. 3D colony formation assay. **a** Representative bright-field image of 3D colonies generated from PIC hydrogels or Matrigel-derived basal or luminal population. Two independent experiments were performed and data was presented for one of the experiments. Every experiment was performed with three gel samples per experimental group (PIC-1, 0.2 wt-%, 63 μM RGD, *G'* = 0.32 kPa; PIC-2,0.8 wt-%, 0 μM RGD, *G'* = 2.4 kPa; PIC-3, 0.8 wt-%, 63 μM RGD, *G'* = 2.3 kPa; PIC-4, 0.8 wt-%, 252 μM RGD, *G'* = 2.3 kPa; and Matrigel). Scale bar, 250 μm. **b** Quantification of basal/luminal population-derived 3D colony numbers in PIC hydrogels or Matrigel. Only colony with diameter above 20 μm was counted. Statistics: NS, not significant. ** P<0.01, *** P<0.001, one-way ANOVA followed by Tukey's multiple comparisons test.

a

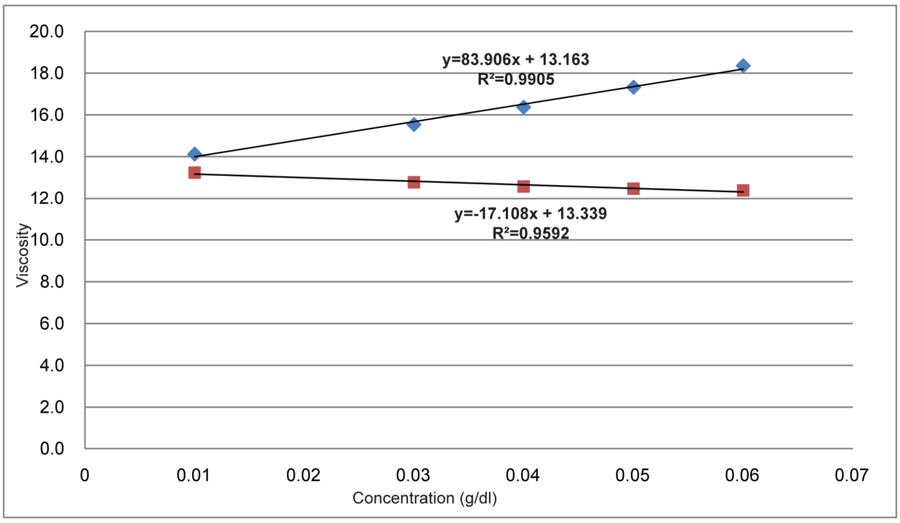

y=83.906x + 13.163
R²=0.9905
y=-17.108x + 13.339
R²=0.9592
Viscosity
20.0
18.0
16.0
14.0
12.0
10.0
8.0
6.0
4.0
2.0
0.0
0
0.01
0.02
0.03
0.04
0.05
0.06
0.07
Concentration (g/dl)

b

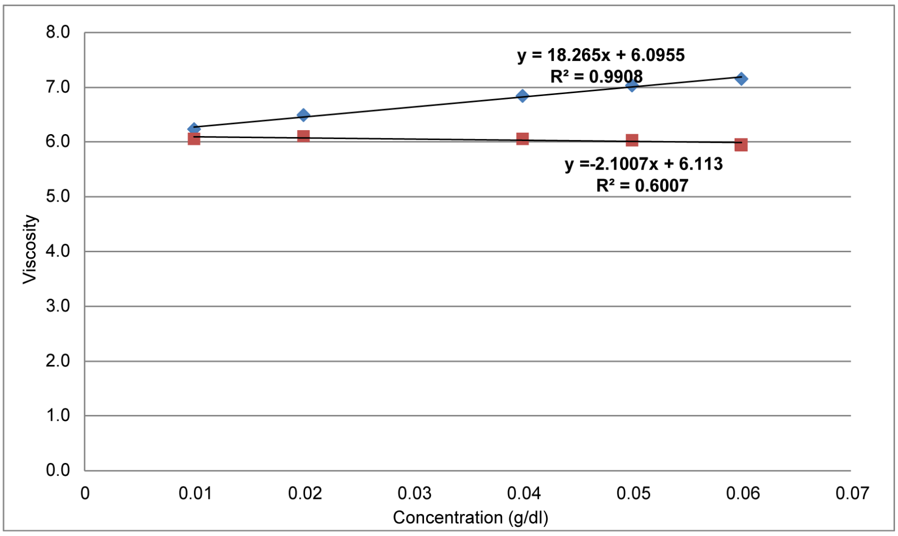

y = 18.265x + 6.0955
R² = 0.9908
y =-2.1007x + 6.113
R² = 0.6007
Viscosity
8.0
7.0
6.0
5.0
4.0
3.0
2.0
1.0
0.0
0
0.01
0.02
0.03
0.04
0.05
0.06
0.07
Concentration (g/dl)

c

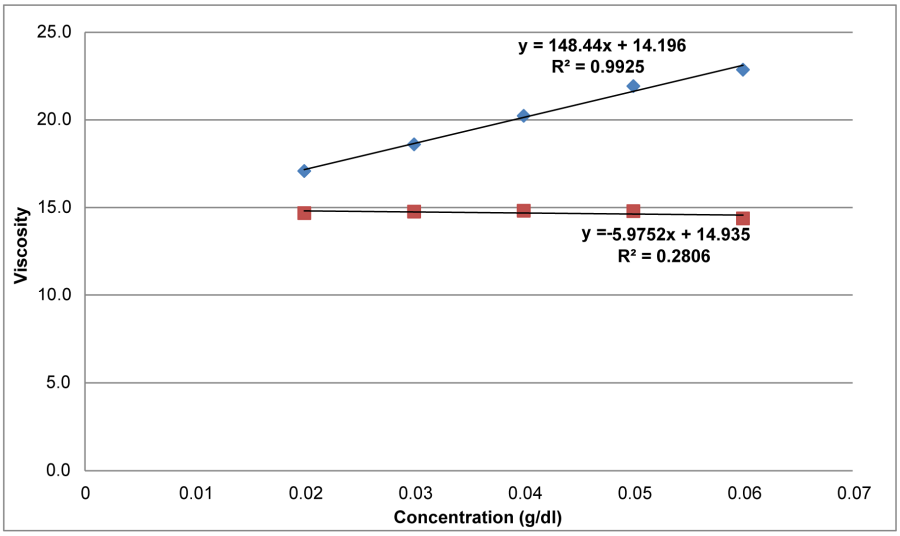

y = 148.44x + 14.196
R² = 0.9925
y =-5.9752x + 14.935
R² = 0.2806
Viscosity
25.0
20.0
15.0
10.0
5.0
0.0
0
0.01
0.02
0.03
0.04
0.05
0.06
0.07
Concentration (g/dl)

Figure S9. Viscometry of PIC polymer solutions: 1k (a), 3k (b) and 5k (c). The related molecular weight M_v = 322 kg mol^{-1} (1K), 502 kg mol^{-1} (3K), and 530 kg mol^{-1} (5K) were calculated based on polymer concentration and its viscosity data on PIC-azide in acetonitrile solutions.

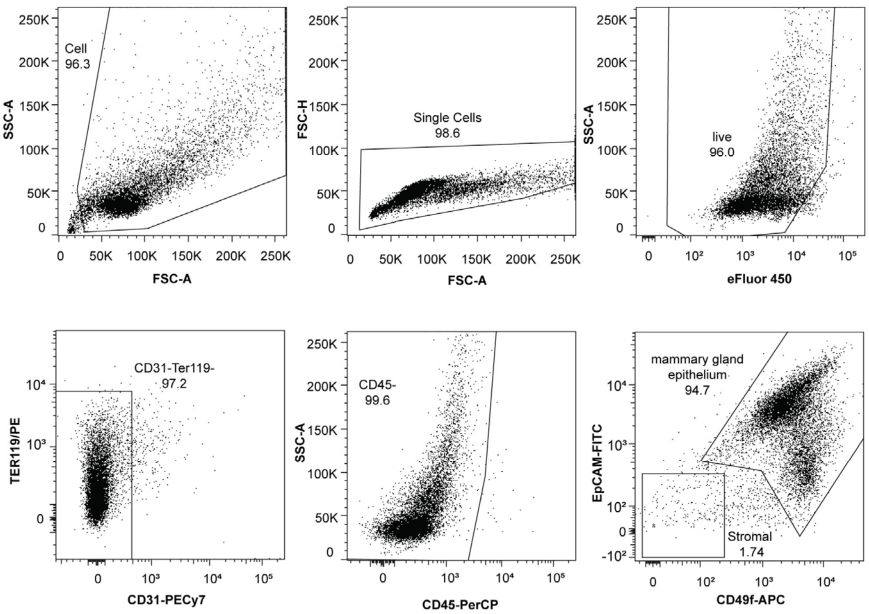

Figure S10. Gating strategy of flow cytometry analysis for segregating mammary epithelium (eFluor450/CD31-/TER119-/CD45-) from erythrocytes, dead cells, endothelial and immune cells.
